# Acute Effect of Positive Airway Pressure on Heart Rate Variability in Obstructive Sleep Apnea

**DOI:** 10.3390/jcm12247606

**Published:** 2023-12-10

**Authors:** Ji Hye Shin, Min Ji Song, Ji Hyun Kim

**Affiliations:** Department of Neurology, Korea University Guro Hospital, Korea University College of Medicine, Seoul 08308, Republic of Korea; snjih@naver.com (J.H.S.); songminji1034@gmail.com (M.J.S.)

**Keywords:** obstructive sleep apnea, heart rate variability, continuous positive airway pressure

## Abstract

Autonomic dysregulation is associated with cardiovascular consequences in obstructive sleep apnea (OSA). This study aimed to investigate the effect of acute continuous positive airway pressure (CPAP) treatment on autonomic activity and to identify factors contributing to heart rate variability (HRV) changes in OSA. Frequency domain HRV parameters were calculated and compared between the baseline polysomnography and during the CPAP titration in 402 patients with moderate to severe OSA. There were significant reductions in total power, very low-frequency band power, low-frequency band power, and high-frequency band power during the CPAP titration as compared to the baseline polysomnography. This tendency was pronounced in male patients with severe OSA. Multivariate analysis found that changes in the apnea-hypopnea index and oxygen saturation were significantly associated with changes in sympathetic and parasympathetic activity, respectively. This study demonstrated that HRV parameters significantly changed during the CPAP titration, indicating a beneficial effect of CPAP in the restoration of sympathetic and parasympathetic hyperactivity in OSA. Prospective longitudinal studies should determine whether long-term CPAP treatment aids in maintaining the long-lasting improvement of the autonomic functions, thereby contributing to the prevention of cardiovascular and cerebrovascular diseases in patients with OSA.

## 1. Introduction

Recent epidemiological studies have indicated that obstructive sleep apnea (OSA) is an independent risk factor for stroke, hypertension, atrial fibrillation, coronary artery disease, heart failure, and sudden cardiac death [1,2]. The exact mechanism underlying this link is not fully elucidated; however, recent studies have shown that OSA patients frequently exhibit an excessive activation of the sympathetic nervous system, suggesting a role of disturbed cardiac autonomic regulation in the development of cardiovascular and cerebrovascular diseases [3,4]. Frequent respiratory events during sleep, including intermittent hypoxemia, hypercapnia, intrathoracic pressure change, and recurrent arousals, alter the sympathovagal balance towards sympathetic hyperactivity [5,6]. Meanwhile, the impact of OSA on parasympathetic modulation during sleep remains controversial [7].

It has been proposed that both sympathetic and parasympathetic systems are physiologically co-activated in individuals with OSA [8]. The interaction between these two systems in relation to the heart may not always follow a reciprocal pattern [9]. A decrease in the arterial partial pressure of oxygen or an increase in the arterial partial pressure of carbon dioxide may lead to simultaneous activation of sympathetic and parasympathetic activities, which disrupts the reciprocal interaction between the two activities [9]. Respiratory collapse, hypoxia, and recurrent arousals could lead to parasympathetic hyperactivity during apneic events, followed by sympathetic hyperactivity in patients with OSA. The initial stage of apneic events, characterized by resistive breathing without accompanying hypoxemia, may exhibit a heightened parasympathetic tone [10]. Increased inspiratory efforts give rise to a reduction in parasympathetic tone and an elevation in sympathetic tone at the termination of apnea.

Heart rate is regulated by sympathetic and parasympathetic activities, and heart rate variability (HRV) serves as a simple and useful method for assessing cardiac autonomic functions [11]. Previous studies analyzing HRV have shown alterations in sympathetic or parasympathetic activity in patients with OSA [12,13,14,15,16,17]. Both low-frequency power (LF) and LF-to-high-frequency power (HF) ratios consistently increased in OSA patients compared to healthy controls, pointing to a shift in the sympathovagal balance toward sympathetic hyperactivity [12,13,14,15,18]. Meanwhile, other studies have yielded somewhat inconsistent findings with regard to HF, an HRV parameter reflecting parasympathetic activity: decreased HF [8,18,19,20,21] or increased HF [12,22] in OSA patients when compared to controls.

There is mounting evidence that continuous positive airway pressure (CPAP) treatment improves HRV in patients with OSA [18,21,23,24,25,26,27,28]. A significant decrease in sympathetic hyperactivity, as assessed by HRV, was demonstrated both on the first night of CPAP titration [21,25,27] and after long-term CPAP treatment [18,21,24,28]. However, several studies reported no significant change in sympathetic activity after CPAP treatment [29,30,31]. In addition, the effect of CPAP treatment on parasympathetic activity has also yielded inconsistent results across the studies: increased HF [18,21,27,31], decreased HF [23,24,26], and unaltered HF [29,30,32]. The inconsistent findings observed in prior studies regarding HRV changes following CPAP treatment might be, in part, attributed to the recruitment of a relatively small number of patients.

This present study aimed (1) to determine whether acute CPAP treatment can modify HRV alterations and restore abnormal sympathetic and parasympathetic activity, and (2) to identify the independent factors affecting HRV alterations while controlling potential confounding factors. We hypothesize that not only LF reflecting sympathetic activity, but also HF reflecting parasympathetic activity is decreased during acute CPAP treatment, by way of correcting hypoxemia in patients with OSA.

## 2. Materials and Methods

### 2.1. Study Population

We retrospectively studied 548 patients with moderate to severe OSA who underwent overnight polysomnography (PSG) and consecutive PSG for CPAP titration at the sleep center of the Korea University Guro Hospital between 2011 to 2022. Patients with a history of stroke, myocardial infarction, atrial fibrillation, angina pectoris, and hyperthyroidism were excluded. Patients with other sleep disorders that can influence the autonomic function, such as rapid eye movement (REM) sleep behavior disorder, restless legs syndrome, and narcolepsy [33], were further excluded from the analysis. All patients included in the final analysis did not take any anticholinergic (e.g., atropine, benztropine, trihexyphenidyl), sympathomimetic (e.g., epinephrine, phenylephrine, isoproterenol, salbutamol) or parasympathomimetic (e.g., bethanechol, pilocarpine) medications. All patients completed the Pittsburgh Sleep Quality Index, Epworth Sleepiness Scale, and the SF-36 quality of life questionnaires. This study was approved by the local ethic committee of the Korea University Guro Hospital (IRB No. 2014GR0180).

### 2.2. Polysomnography

An overnight PSG recording was conducted using the Embla N7000 system (Natus Medical Inc., Pleasanton, CA, USA). Electroencephalography was recorded using four pairs of leads (C4-A1, C3-A2, O2-A1, and O1-A2) with two pairs of electro-oculographic leads. Electromyographic leads were attached to the tibialis anterior and submentalis muscles. Airflow was continuously monitored using a thermistor and a nasal pressure cannula, and arterial oxygen saturation was measured via a pulse oximeter. Respiratory motions were tracked with the use of inductive plethysmographic belts wrapped tightly around both the abdomen and chest.

Sleep stages and events were evaluated and scored based on the guidelines established by the American Academy of Sleep Medicine [34]. The parameters related to sleep architecture were the following: total sleep time, time spent in bed, sleep latency, wake after sleep onset, stage 1 sleep, stage 2 sleep, slow-wave sleep, REM sleep, sleep efficiency, and arousal. The arousal index (AI) was determined as the total number of arousals per hour, calculated by adding the periodic limb movement AI, apnea-hypopnea AI, and spontaneous AI. The oxygen desaturation index was determined by counting the number of events in which oxygen saturation decreased by 3% or more per hour. Apnea was characterized as a reduction in airflow ≥90% of baseline for a minimum duration of 10 s. Hypopnea was characterized by a reduction in airflow ≥30% of baseline for a minimum duration of 10 s, accompanied by either an arousal or a 3% decrease in oxygen saturation [34]. The apnea-hypopnea index (AHI) was determined by the number of apnea-hypopnea events per hour of recorded sleep time. Patients were classified into mild OSA (5 ≤ AHI < 15), moderate OSA (15 ≤ AHI < 30), and severe OSA (AHI ≥ 30) groups.

### 2.3. Heart Rate Variability

The electrocardiography data retrieved from the whole overnight PSG recording were visually inspected for quality and reliability, and then used for HRV analysis. Artefacts and ectopic beats were automatically removed, and only normal-to-normal beats were chosen for HRV analysis [35]. The assessment of HRV comprises time domain and frequency domain analyses. The time domain HRV parameters quantify the amount of variability in beat-to-beat intervals, and are influenced by both sympathetic and parasympathetic activities. Consequently, these parameters cannot differentiate the specific roles of sympathetic and parasympathetic activities in autonomic nervous system functions [11]. The frequency domain HRV parameters are measured by using the fast Fourier transform and classified into different frequency ranges with associated spectral powers. This process offers information regarding specific alterations in sympathetic and parasympathetic activity [11]. We focused on the frequency domain parameters to better evaluate autonomic function changes by distinguishing between sympathetic and parasympathetic activity. The following parameters were calculated for spectral analysis executed in RemLogic software (Version 2.0; Embla Co., Broomfield, USA): (1) total power spectrum (TP), (2) high-frequency band power (HF; 0.15–0.40 Hz), (3) low-frequency band power (LF; 0.04–0.15 Hz), and (4) very low-frequency band power (VLF; 0.0033–0.04 Hz). HF and LF are generally accepted to reflect parasympathetic activity and baroreflex-mediated sympathetic activity, respectively [35]. The LF/HF ratio is recognized as the representative index of sympathetic to parasympathetic (sympathovagal) balance with a higher LF/HF indicating sympathetic hyperactivity [35]. The TP and VLF, overall, reflect a measure of autonomic function and parasympathetic activity, respectively.

### 2.4. Statistical Analysis

Descriptive statistics illustrated the baseline characteristics and PSG parameters of the study population. The Kolmogorov–Smirnov test was first conducted to assess normality, and all variables were found not to follow a normal distribution (*p* < 0.05). Within-group comparisons of HRV parameters, changes between the baseline PSG and during the CPAP titration were made using the Wilcoxon signed-rank test. This analysis was repeated for subgroups related to sex and disease severity using the Wilcoxon signed-rank test: male-severe, male-moderate, female-severe, and female-moderate groups. Next, overall differences in each HRV parameter among the four groups were tested using the Kruskal–Wallis test. Pairwise comparisons of HRV parameters were then performed by using Mann–Whitney U test, followed by the Bonferroni correction (*p* < 0.008 [0.05/6]).

Multivariate linear regression analysis was performed to assess significant associations between the independent variables (e.g., demographics, changes in PSG variables) and dependent variables (changes in frequency domain HRV parameters). The effect of multicollinearity between the independent variables was assessed using variance inflation factors. Only variables with a variance inflation factor < 5 entered into multivariate linear regression analysis as independent variables. The statistical significance was set at *p* < 0.05 in all tests. Statistical analyses were performed with the Statistical Package for the Social Sciences software (Version 26.0; IBM Corp., Armonk, New York, NY, USA).

## 3. Results

**Figure 1:**
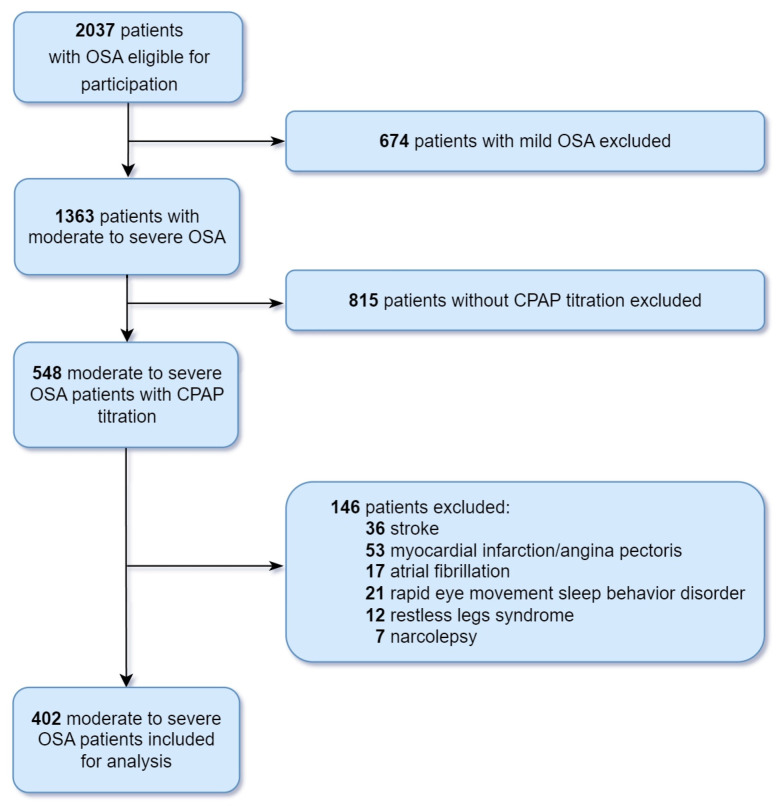
Flow diagram depicting the patient selection process. Abbreviations: CPAP, continuous positive airway pressure; OSA, obstructive sleep apnea.

Of 548 patients with moderate to severe OSA, 146 were excluded due to medical history of stroke (*n* = 36), angina pectoris (*n* = 53), atrial fibrillation (*n* = 17), REM sleep behavior disorder (*n* = 21), restless legs syndrome (*n* = 12), and narcolepsy (*n* = 7). This resulted in a final study sample of 402 patients (Figure 1). Demographics, clinical characteristics, sleep questionnaires, and PSG parameters of the 402 patients (320 males and 82 females, 132 moderate OSA and 270 severe OSA) are summarized in Table 1.

Table 2 summarizes the details of the PSG parameters and HRV parameters of the baseline PSG and during the CPAP titration as well as their corresponding statistical results. Waking after sleep onset, the N1 sleep, AHI, oxygen desaturation index, and AI significantly decreased during the CPAP titration when compared to the baseline PSG (all *p* < 0.001). Sleep efficiency, N2 sleep, N3 sleep, REM sleep, and mean arterial oxygen saturation significantly increased during the CPAP titration when compared to the baseline PSG (all *p* < 0.001). The following frequency domain HRV parameters significantly decreased during the CPAP titration as compared to the baseline PSG: TP (*p* < 0.001), VLF (*p* < 0.001), LF (*p* = 0.001), and HF (*p* < 0.001). There was no significant change in LF/HF ratio between the baseline PSG and during the CPAP titration (*p* = 0.978).

Results of the subgroup analyses related to sex and disease severity are summarized in Table 3. In the male-severe group (*n* = 230), all frequency domain HRV parameters significantly decreased during the CPAP titration compared to the baseline PSG: TP (*p* < 0.001), VLF (*p* < 0.001), LF (*p* < 0.001), and HF (*p* = 0.001). In the male-moderate group (*n* = 90), HF (*p* = 0.038) significantly decreased, and LF/HF ratio (*p* = 0.041) significantly increased during the CPAP titration compared to the baseline PSG. There were no significant changes in all HRV parameters between the baseline PSG and during the CPAP titration in the female-severe (*n* = 40) and female-moderate (*n* = 42) groups (all *p* > 0.05). Results of between-group comparisons of changes in HRV parameters are summarized in Table 4. The changes in TP, VLF, and LF during the CPAP titration in the male-severe group were larger than those in the male-moderate, female-severe, and female-moderate groups (*p* < 0.008). The change in HF was not different among the four groups.

Table 5 summarizes the results of multivariate linear regression analysis for significant associations between the independent variables and changes in frequency domain HRV parameters. Changes in TP significantly correlated with changes in AHI (*p* = 0.025) and BMI (*p* = 0.042). Changes in VLF significantly correlated with changes in AHI (*p* = 0.024) and age (*p* = 0.024). Changes in LF significantly correlated with changes in AHI (*p* = 0.044) and BMI (*p* = 0.006). Changes in HF significantly correlated with changes in mean arterial oxygen saturation (*p* < 0.001) and age (*p* = 0.032).

## 4. Discussion

In this present study, we aimed to determine whether acute CPAP treatment can improve HRV in patients with moderate to severe OSA. The main findings are that HRV parameters significantly changed during the CPAP titration, indicating an important role of CPAP treatment in restoration of sympathetic and parasympathetic hyperactivity in patients with OSA. This tendency was pronounced in male patients with severe OSA. Multivariate linear regression analysis found that changes in AHI and mean arterial oxygen saturation are significantly associated with changes in sympathetic and parasympathetic activity, respectively.

Previous studies have examined the effect of CPAP on sympathetic hyperactivity in OSA patients, and repeatedly demonstrated a significant reduction in LF during both acute CPAP treatment [21,25,27] and long-term CPAP treatment [18,21,24]. In a prospective longitudinal study, HRV was analyzed during the first night of CPAP treatment and after 2 years of CPAP treatment in 30 patients with moderate to severe OSA [21]. Both acute and long-term CPAP treatment decreased the LF during non-REM and REM sleep, indicating a beneficial role of CPAP treatment on sympathetic hyperactivity in OSA [21]. A meta-analysis including 11 studies found a significant decrease in LF in OSA patients receiving CPAP treatment for a duration ranging from 1 month to over 12 months, suggesting that long-term CPAP treatment potentially improves cardiac autonomic function [36]. Another meta-analysis including 17 studies found a tendency toward a small decrease in sympathetic measures of HRV with various treatments for OSA [7]. Heterogeneity in HRV measures (LF, normalized LF, and LF/HF ratio) and treatment modalities (e.g., CPAP, mandibular advancement device, and oral jaw-positioning appliance) may introduce potential confounding factors in the results. In line with the aforementioned studies, our large-scale study demonstrated a significant reduction in LF during acute CPAP treatment compared to the baseline. This trend of significantly reduced LF was pronounced in male patients with severe OSA. Similar to our study, 16 patients with severe OSA showed a significant reduction in LF during acute CPAP treatment in both REM and non-REM sleep, while 14 patients with moderate OSA did not [21], implying that the increased OSA severity may lead to a heightened effectiveness of acute CPAP treatment in restoring normal sympathetic activity. Taken together, CPAP treatment may have the potential to improve sympathetic hyperactivity observed in patients with OSA. However, the finding of reduced LF following CPAP treatment was not replicated in previous studies investigating the same paradigm. No statistically significant change in LF was observed in moderate to severe OSA patients receiving 3 months CPAP treatment [29,30].

It is generally considered that CPAP treatment decreases sympathetic activity while increasing parasympathetic activity, exhibiting opposing yet complementary effects on these two activities. The impact of CPAP treatment on parasympathetic activity has also been examined in OSA, with inconsistent results across the studies. Several currently available studies have reported a significant increase in HF (i.e., increased parasympathetic activity) during both acute CPAP treatment [21,27] and long-term CPAP treatment [18,21,31]. However, no significant change in HF was found in moderate to severe OSA patients receiving 3 months CPAP treatment, compared to the baseline [30,32]. There was no overall change in parasympathetic measures of HRV following CPAP treatment in a meta-analysis including 19 studies [7]. This discrepancy warrants the need for a prospective longitudinal study to further investigate the issue of HF alteration with CPAP treatment.

The sympathetic nervous system, known as the “fight or flight” system, is associated with high arousal and activity, while the parasympathetic nervous system is regarded as the “rest and digest” system, and is linked to relaxation and recovery. Sympathetic and parasympathetic activities appear to have contrasting and reciprocal roles, implying that when one activity increases, the other activity typically decreases. Meanwhile, mild hypoxia causes a co-activation of both cardiac sympathetic and vagal nerve activities [9]. Upper airway obstruction observed in OSA patients may increase parasympathetic activity and, in turn, cause hypoxia, resulting in an abrupt increase in sympathetic activity [10,28]. In the healthy population, it is well documented that increased parasympathetic activity and decreased sympathetic activity are typically observed during non-REM sleep, whereas the opposite occurs during REM sleep [37]. Patients with OSA exhibit a similar pattern, although they tend to show more pronounced parasympathetic activity during sleep [22,28]. Moderate to severe OSA patients showed greater parasympathetic activation during non-REM sleep compared to mild OSA patients and non-OSA controls, indicating a positive association between apnea severity and parasympathetic activity [38]. An increase in parasympathetic activity may be linked to an increase in hydraulic pressure, which could be initiated by the carotid aortic chemoreceptor in response to hypoxemia [38]. Patients with OSA displayed a noteworthy decrease in HF with CPAP treatment compared to their baseline measurements [23,25]. Our finding of a significant decrease in HF during CPAP titration in moderate to severe OSA patients accords well with those of previous studies, suggesting that CPAP treatment may improve HRV by way of reducing not only sympathetic activity but also parasympathetic activity. 

It is widely recognized that OSA is linked to an increased incidence of cardiovascular and cerebrovascular diseases [39,40]. Previous studies provided compelling evidence that untreated moderate to severe OSA in patients with established coronary disease [41] and heart failure [42] is associated with increased cardiovascular and cerebrovascular morbidity and mortality. Data from small-scale trials provide evidence that treatment of OSA with CPAP improves cardiovascular outcomes such as lowering blood pressure and reducing incidence of repeat revascularization, myocardial infarction, and stroke [39,43]. A long-term observational study clearly demonstrated that the incidence of fatal and non-fatal cardiovascular and cerebrovascular events in untreated patients with severe OSA is significantly higher than that in untreated mild-moderate OSA patients and in healthy participants who were individually matched for BMI and age [40]. Notably, treatment with CPAP significantly reduced cardiovascular and cerebrovascular risks in patients with severe OSA, and this reduction was not significantly different from that observed in healthy participants [40]. It is previously assumed that long-term CPAP treatment partially reduces sympathetic hyperactivity and restores the impaired sympathovagal balance in OSA patients [44], which may be one of the potential mechanisms in preventing the development of cardiovascular diseases [45]. Our finding that acute CPAP treatment reduced both sympathetic and parasympathetic activity provides a theoretical framework supporting the notion that CPAP treatment may contribute to the reduction of cardiovascular and cerebrovascular diseases. 

This study has several strengths. First, a substantial number of patients were enrolled, ensuring statistical reliability due to the large sample size. Second, we utilized multivariate linear regression analysis to identify the variables associated with the observed HRV differences. Changes in LF were closely related to changes in AHI, suggesting that AHI is an important contributing factor for changes in sympathetic activity induced by CPAP treatment in OSA. We also found a significant correlation between changes in HF and changes in mean arterial oxygen saturation, implying that hypoxia, independent of apnea, is associated with alterations in parasympathetic activity. This study has also several potential limitations that should be addressed. First, the inclusion of patients who were referred to a university-affiliated hospital may introduce a selection bias, limiting the generalizability of the findings to the entire OSA population. Second, we did not exclude patients with comorbidities that may affect autonomic functions, such as hypertension, diabetes mellitus, or obesity. Nevertheless, a within-subject design employed in this study can help mitigate the influence of comorbidity issue on autonomic measurements. Third, HRV alterations observed during the CPAP titration may result from changes in venous return secondary to the substantial intrathoracic pressure fluctuations during the apneic events rather than the changes in cardiovascular control mechanisms. Thus, the influence of CPAP treatment on autonomic alterations in OSA patients may be better assessed by comparing baseline HRV parameters with those obtained after several weeks of CPAP treatment, rather than those obtained during acute CPAP treatment. Last, technical limitations in HRV analysis may arise from the use of HRV data obtained from the entire overnight PSG recording without excluding arousals and apneas, which can alter HRV parameters. 

## 5. Conclusions

In conclusion, we have shown that acute CPAP treatment restores sympathetic and parasympathetic hyperactivity, suggesting a beneficial effect of CPAP on autonomic functions in patients with moderate to severe OSA. Given the close relationship between autonomic alterations and increased risks of cardiovascular and cerebrovascular diseases in OSA, prospective longitudinal studies are warranted to determine whether long-term CPAP treatment aids in maintaining the longstanding improvement of the autonomic functions, thereby contributing to the prevention of such consequences.

## Figures and Tables

**Table 1:** Clinical characteristics of the study population.

Clinical Characteristics	
Male: Female, *n* (%)	320 (79.6%): 82 (20.4%)
Age, years	52.0 ± 11.8 (range = 16–81)
BMI (kg/m^2^)	28.8 ± 4.4 (range = 19–48.9)
Moderate: Severe OSA, *n* (%)	132 (32.8%): 270 (67.2%)
Hypertension, *n* (%)	228 (56.7%)
Diabetes mellitus, *n* (%)	77 (19.2%)
Current smoker, *n* (%)	91 (22.6%)
ESS score	8.3 ± 4.6 (range = 0–24)
PSQI	7.5 ± 4.6 (range = 0–22)
SF-36	68.2 ± 19.4 (range = 15–98)

Abbreviations: BMI, body mass index; ESS, Epworth Sleepiness Scale score; PSQI, Pittsburgh Sleep Quality Index; SF-36, Medical Outcome Study 36-item short-form health survey. Values are expressed as mean ± standard deviations or number (percentage).

**Table 2:** PSG parameters and frequency domain HRV parameters of the baseline PSG and during the CPAP titration.

PSG Parameters				
	Baseline	During CPAP	*p*	
Total sleep time (min)	349.0 ± 48.8	349.8 ± 46.5	0.791	
Sleep efficiency (%)	84.6 ± 10.7	86.7 ± 10.1	<0.001	
WASO (min)	53.3 ± 40.9	45.1 ± 38.1	<0.001	
N1 (%)	127.2 ± 56.3	68.2 ± 33.5	<0.001	
N2 (%)	126.5 ± 88.7	162.5 ± 45.2	<0.001	
N3 (%)	11.3 ± 19.2	15.6 ± 23.5	<0.001	
REM (min)	86.7 ± 33.3	103.5 ± 37.6	<0.001	
AHI	43.9 ± 22.0	2.6 ± 3.1	<0.001	
REM-AHI	47.4 ± 22.1			
NREM-AHI	42.0 ± 24.6			
ODI	41.0 ± 22.3	3.5 ± 5.8	<0.001	
AI	50.7 ± 22.7	15.1 ± 12.4	<0.001	
Mean SaO_2_	93.7 ± 2.5	95.8 ± 1.2	<0.001	
**Frequency domain HRV parameters**				
	Baseline	During CPAP	Difference	*p*
TP (ms^2^)	39,836 ± 20,001	35,672 ± 17,055	4164 ± 2946	<0.001
VLF (ms^2^)	21,184 ± 12,586	18,494 ± 10,006	2690 ± 2580	<0.001
LF (ms^2^)	12,472 ± 7636	11,560 ± 7157	912 ± 479	0.001
HF (ms^2^)	5380 ± 2917	4920 ± 2507	460 ± 410	<0.001
LF/HF ratio	2.9 ± 2.3	2.8 ± 2.2	0.1 ± 0.1	0.978

Abbreviations: AHI, apnea-hypopnea index; AI, arousal index; HF, high-frequency power; LF, low-frequency power; NREM, non-REM; ODI, oxygen desaturation index; REM, rapid eye movement; SaO_2_, arterial oxygen saturation; TP, total power; VLF, very low-frequency power; WASO, waking after sleep onset. Values are expressed as mean ± standard deviations.

**Table 3:** Results of the subgroup analysis related to sex and disease severity of changes in frequency domain HRV parameters between the baseline PSG and during the CPAP titration.

PSG Parameters						
	Male-severe (*n* = 230)			Male-moderate (*n* = 90)		
	Baseline	During CPAP	*p*	Baseline	During CPAP	*p*
TP (ms^2^)	43,682 ± 20,907	37,445 ± 17,293	<0.001	40,712 ± 18,397	40,096 ± 16,776	0.565
VLF (ms^2^)	23,781 ± 13,638	19,760 ± 10,444	<0.001	20,958 ± 10,437	20,306 ± 9008	0.478
LF (ms^2^)	13,742 ± 7651	12,115 ± 6924	<0.001	13,170 ± 7929	13,695 ± 8154	0.480
HF (ms^2^)	5311 ± 13,638	4869 ± 2442	0.001	5905 ± 3224	5450 ± 2942	0.038
LF/HF ratio	3.2 ± 2.4	2.9 ± 2.0	0.283	2.8 ± 2.2	3.2 ± 2.9	0.041
	Female-severe (*n* = 40)			Female-moderate (*n* = 42)		
	Baseline	During CPAP	*p*	Baseline	During CPAP	*p*
TP (ms^2^)	27,359 ± 15,634	25,169 ± 12,854	0.221	28,781 ± 11,920	26,484 ± 12,322	0.149
VLF (ms^2^)	13,499 ± 9157	12,422 ± 7781	0.307	14,765 ± 7511	13,458 ± 7630	0.123
LF (ms^2^)	8292 ± 6821	7656 ± 5592	0.476	7995 ± 3860	7660 ± 4128	0.520
HF (ms^2^)	4805 ± 3320	4318 ± 2250	0.436	5177 ± 2280	4637 ± 1873	0.083
LF/HF ratio	2.5 ± 2.7	2.2 ± 1.6	0.610	1.8 ± 1.2	1.7 ± 1.0	0.846

Abbreviations: HF, high-frequency power; LF, low-frequency power; TP, total power; VLF, very low-frequency power. Values are expressed as mean ± standard deviations.

**Table 4:** Results of between-group comparisons of changes in frequency domain HRV parameters.

	Male-Severe (*n* = 230)	Male-Moderate (*n* = 90)	Female-Severe (*n* = 40)	Female-Moderate (*n* = 42)	Overall *p*
∆TP (ms^2^)	6237 ± 17,279 ^a^	615 ± 12,870	2190 ± 9648	2297 ± 9812	0.010
∆VLF (ms^2^)	4020 ± 11,565 ^a^	652 ± 7304	1076 ± 6119	1307 ± 5669	0.013
∆LF (ms^2^)	1627 ± 6732 ^a^	−525 ± 5611	636 ± 5261	335 ± 3266	0.010
∆HF (ms^2^)	441 ± 2239	455 ± 2285	486 ± 2116	540 ± 1749	0.919

Abbreviations: HF, high-frequency power; LF, low-frequency power; TP, total power; VLF, very low-frequency power; ∆, average amount of change. ^a^ *p* < 0.008 compared with male-moderate, female-severe, and female-moderate groups. Values are expressed as mean ± standard deviations.

**Table 5:** Results of multivariate linear regression analysis for variables associated with changes in HRV parameters.

Multivariate Linear Regression Analysis				
Independent variables	∆VLF	∆LF	∆HF	∆TP
Age	0.129 *	0.052	–0.119 *	0.086
Sex	–0.078	–0.015	0.007	–0.056
BMI	0.061	0.150 **	0.084	0.112 *
∆AHI	0.188 *	0.166 *	–0.025	0.186 *
∆AI	–0.070	–0.047	–0.038	–0.069
∆mean SaO_2_	–0.021	–0.038	0.312 ***	0.013

Abbreviations: AHI, apnea-hypopnea index; AI, arousal index; BMI, body mass index; HF, high-frequency power; LF, low-frequency power; SaO_2_, arterial oxygen saturation; TP, total power; VLF, very low-frequency power; ∆, average amount of change. Values are expressed as standardized beta coefficients; * *p* < 0.05, ** *p* < 0.01, *** *p* < 0.001.

## Data Availability

The dataset used and analyzed in the current study is available from the corresponding author on reasonable request.
